# Definitive chemoradiation in patients with inoperable oesophageal carcinoma

**DOI:** 10.1038/sj.bjc.6601461

**Published:** 2004-01-06

**Authors:** T D L Crosby, A E Brewster, A Borley, L Perschky, P Kehagioglou, J Court, T S Maughan

**Affiliations:** 1 Velindre Cancer Centre, Velindre Place, Cardiff CF14 2TL, UK; 2 University of Wales College of Medicine, Heath Park, Cardiff CF14 4XN, UK

**Keywords:** definitive chemoradiation, inoperable, oesophageal cancer

## Abstract

We performed a retrospective study of 90 consecutive cases with inoperable carcinoma of the oesophagus treated with definitive chemoradiation at a single cancer centre between 1995 and 2002. For the last 4 years, 73 patients have received therapy according to an agreed protocol. This outpatient-based regimen involves four cycles of chemotherapy, cycles 3 and 4 given concurrently with 50 Gy external beam radiotherapy (XRT) delivered in 25 fractions over 5 weeks. Cisplatin 60 mg m^−2^ day^−1^ is given every 3 weeks together with continuous infusional 5-fluorouracil 300 mg m^−2^ day^−1^, reduced to 225 mg m^−2^ day^−1^ during the XRT. In all, 45 (50%) patients suffered one or more WHO grade 3/4 toxicity, grade 3 in 93% cases. Patients received more than 90% of the planned chemoradiation schedule. The median overall survival was 26 (15, >96) months, 51% (41, 64) and 26% (13, 52) surviving 2 and 5 years, respectively. Advanced stage, particularly T4 disease, was associated with a worse prognosis. Patients considered not suitable for surgery for reasons other than their disease, mainly co-morbidity, had a significantly better outcome, median survival 40 (26, >96) months, 2- and 5-year survivals 67% (54, 84) and 32% (13, 79), respectively (*P*<0.001). This schedule is a feasible, tolerable and effective treatment for patients with oesophageal cancer considered unsuitable for surgery.

Carcinoma of the oesophagus is becoming more common in Europe and North America. In the UK, there are 7000 new cases per year, the eighth most common malignancy, and in some regions it has become more common than gastric cancer (Scottish Audit Group, 2002). Despite difficulties with categorising tumours at the gastro-oesophageal junction, there has clearly been a significant increase in the number of adenocarcinomas occurring in the lower oesophagus and proximal stomach, probably as a consequence of gastro-oesophageal reflux disease. Given that the presenting symptoms commonly indicate advanced disease, it is not surprising that the long-term outcome remains poor, just 10% of patients surviving 5 years after diagnosis.

In the UK, if the disease appears resectable and patients are sufficiently fit, surgery remains the mainstay of therapy. However, despite better staging and improving perioperative care, the outcome from this treatment remains poor, with 30–45% patients surviving 2 years after radical resection (Kelsen *et al*, 1998; Medical Research Council Oesophageal Cancer Working Group, 2002). The proportion of patients for whom this treatment is selected varies in different countries from 25 to 45% (Fok *et al*, 1994; Coia *et al*, 2000; Pye *et al*, 2001). This complex surgery is challenging for both the surgeon and the patient; between 5 and 10% will die as a result of an oesophagectomy and 30–40% will suffer significant postoperative morbidity. In addition, those patients who relapse within 2 years seldom regain the quality of life they previously experienced (Blazeby *et al*, 2000). Some improvement in these figures may be possible with adjuvant therapy (Walsh *et al*, 1996; Medical Research Council Oesophageal Cancer Working Group, 2002) and centralisation of services (NHS executive, 2001), but it is clear that the majority of patients will not benefit from this treatment.

Radical radiotherapy (RT), or chemoradiation (CRT), has lead to long-term survival in some reported series, though it is not absolutely clear how these patients were selected for nonsurgical therapy (Sykes *et al*, 1998; Cooper *et al*, 1999). Using modern techniques and regimens, these therapies have not been tested as an alternative to surgery in prospective randomised trials, and attempts to do so have been unsuccessful. Although heavily weighted by the results from one trial, a systematic review concludes that concurrent chemoradiation is superior to RT alone, albeit at the expense of increased toxicity (Wong *et al*, 2003).

In South East Wales, since 1998, those patients not suitable for surgery, but where the extent of disease can be covered in a radical radiation field, have been considered for definitive chemoradiotherapy (CRT). Patients were deemed not suitable for surgery because of factors relating to their disease, usually infiltration of tumour into other mediastinal organs, or co-morbidity, making surgery unacceptably hazardous. This study assesses the outcome for this group of patients.

## PATIENTS AND METHODS

This is a retrospective study of 90 consecutive patients treated between 1 March 1995 and 1 October 2002, who received definitive CRT at the Velindre Cancer Centre. One patient was diagnosed and treated in 1995 and three in 1997, and, based on encouraging early results at our centre and elsewhere, a further 86 patients were treated between 1998 and 2002. All patients were discussed at multidisciplinary meetings attended by one or more specialist gastro-enterologists, radiologists, pathologists, surgeons, oncologists, palliative care and upper GI nurses. Patients were initially seen by the surgical team and counselled regarding the risks and likely success of an oesophagectomy. The reasons for patients not receiving surgery are shown in Table 1

**Table 1:** Reasons given for patients not receiving surgery

	**Number (%)**
*Co-morbidity*	50 (56%)
Cardiac	18 #
Pulmonary	15 #
Performance status	11 #
Other	6 #
	
*Disease*	38 (42%)
T4 (aorta)	16 #
T4 (trachea)	7 #
T4 (pleura)	4 #
Lymphadenopathy	7 #
Other	4 #
	
Patient choice	2 (2%)

In each case, the predominant reason has been accepted.

. Where more than one reason was given, the predominant factor is stated.

All patients were confirmed histologically to have oesophageal carcinoma. Patients had disease starting at or below 17 cm ab oral, and included patients with Siewert types I and II gastro-oesophageal junctional disease (Siewert *et al*, 1998), provided there was less than 3 cm disease below the squamo-columnar junction.

For the purpose of this analysis, definitive CRT was defined by a planned dose of external beam RT of ⩾45 Gy delivered with chemotherapy, at least partly given concurrently. Over time treatment regimens and techniques evolved but for the last 4 years all patients were managed according to an agreed protocol.

All patients had endoscopic evaluation and a CT scan of thorax and upper abdomen, to exclude those with macroscopic metastatic disease. All patients had an endoscopic ultrasound (EUS) to assess locoregional disease, except for six, of whom five had malignant stricturing which precluded EUS evaluation.

Physiological assessment included routine haematological and biochemical assays. Renal function was assessed further by calculating the glomerular filtration rate (GFR) using the EDTA clearance method or using the Cockroft formula, when an EDTA test was carried out if the predicted GFR was less than 60 ml min^−1^ or where there was a 25% rise in the serum creatinine after cisplatin treatment. Patients being considered for surgery underwent echocardiograms or MUGA scans, lung function testing with spirometry and arterial blood gas analysis.

Of the 90 patients, 73 received the current treatment protocol for definitive CRT. This consists of four cycles of cisplatin and 5-fluorouracil (5FU), cycles three and four being given concurrently with 5 weeks of RT. The cisplatin is given at 60 mg m^−2^ (reduced to 60 mg total dose, where GFR <60 mls min^−1^) as a day case on Day 1 of each of the three weekly cycles. Carboplatin (AUC=5) is given instead of cisplatin if the GFR is less than 40 ml min^−1^ or if significant neuro-/nephrotoxicity occurs during treatment. During the neoadjuvant phase of treatment the 5FU is given as a continuous infusion of 300 mg m^−2^ day^−1^, reduced to 225 mg m^−2^ day^−1^ during the RT, via a central venous catheter. Patients are given prophylactic warfarin 1 mg day^−1^.

The RT dose is 50 Gy in 25 fractions given over 5 weeks in two treatment phases. Phase 1 is given with an anterior–posterior parallel pair field arrangement giving 26–30 Gy, according to the normal tissue tolerance of the spinal cord, heart and lungs. The remainder is given using an anterior and two posterior oblique, three-field plan. The dose was prescribed to the ICRU 50 reference point, usually being the mid-plane of opposed fields and central axis intersection point of multiple fields.

The conformal planned target volume (PTV) is the EUS-defined gross tumour volume (GTV), with a 2–3 cm margin in all directions. Both phases are planned early during the neoadjuvant phase of therapy using a 3D Helax planning system. The GTV is drawn directly onto the axial planning images using distances of the defined primary and nodal disease ab oral and to the tracheal bifurcation, taken as a reference point, derived from the EUS. The fields are checked with a barium study in the simulator prior to treatment. Portal films are taken during the first week of each phase of treatment.

Patients are reviewed by an oncologist or dedicated radiographer prior to the first day of the chemotherapy cycle, and weekly during the chemoradiation, noting treatment-related acute toxicities. The intent is to deliver this treatment in the outpatient department, though some patients require admission for ‘hotel’ needs. Enteral or parenteral feeding is not routinely used.

Of the remaining 17 patients, 13 received the same regimen without the neoadjuvant phase of chemotherapy, one patient the above regimen omitting the 5FU, two received four cycles of epirubicin, cisplatin and infusional 5FU prior to chemoradiation and one patient received two cycles of mitomycin C, ifosfamide and cisplatin, concurrently with 50Gy radiation in 20 fractions.

Following the completion of chemoradiation, a CT scan is performed to establish a disease ‘baseline’ after treatment, though comparison with scans prior to treatment are notoriously difficult to interpret. Patients no longer undergo EUS, as this invasive procedure does not accurately stage disease after CRT (Bowrey *et al*, 1999). Thus for the purpose of this study, no attempt was made to document the response rate, other than disease progression, at this stage.

All patients are reviewed clinically at regular intervals, but do not routinely undergo radiological or endoscopic surveillance. This continues until death or for the purpose of this study until 31 January 2003, when all data were censored. Five patients have been lost to follow-up and their data censored at their last clinical review. Survival is calculated from the date of histological diagnosis. The date of recurrence is taken as the date of the confirmatory investigations used, usually CT scan or endoscopy, or the date a clinical diagnosis of disease progression was made in the absence of these.

## STATISTICAL ANALYSIS

The data were collected from the Information System for Clinical Organisations (ISCO) – a database and patient activity system available in all Welsh hospitals, patient notes and the Welsh Cancer Information and Surveillance Unit registry. Data were analysed using SPlus 2000. The outcome was assessed using the Kaplan–Meier and log-rank methods, and contingency tables were analysed using *χ*^2^. The effect of clinical covariates was determined using Cox regression. Summary statistics are quoted with the 95% confidence intervals in parentheses.

## RESULTS

### Patient and disease characteristics

Outcome data were available on all 90 patients who received definitive chemoradiation. The distribution of patient and disease characteristics is shown in Table 2

**Table 2:** Distribution of patient and disease characteristics of 90 consecutive cases with oesophageal cancer treated with definitive chemoradiation between 1995 and 2002

	**Number (%)**
*Age*	
<50	6 (7%)
50–65	44 (49%)
66–75	33 (36%)
>75	7 (8%)
	
*Sex*	
M	49 (54%)
F	41 (46%)
	
*Morphology*	
Adenocarcinoma	42 (47%)
Squamous	43 (48%)
Other	5 (6%)
	
*Stage*	
I/II (T1N0,T2-3N0, T1-2N1)	20 (22%)
III (T3N1, T4N0-1)	54 (60%)
IVa (M1a disease)	16 (18%)
	
*Tumour site*	
Upper one-third	4 (4%)
Middle one-third	37 (41%)
Lower one-third	34 (38%)
Type 1 GO junction	12 (13%)
Type 2 GO junction	3 (3%)

. The mean age of patients at diagnosis was 64 years (range 41–78). In all, 25 patients were over 70 years of age. There was a slight predominance of males. The median follow-up of the surviving patients was 19.6 months as of 31 January 2003 (range 4.7–95.8 months). A total of 45 patients died during the period of study.

There were similar numbers of adenocarcinomas and squamous cancers in this series. In six cases, due to poor differentiation of the tumour, it was not possible to classify the type. In all, 49 tumours (54%) were situated in the lower one-third of the oesophagus or crossed the gastro-oesophageal junction. The average length of the primary tumour was 6.9 cm (range 2–13 cm) and was ⩾8 cm in 22 cases (24%).

### Safety and toxicity

The majority of patients received the chemoradiation schedule as prescribed, the commonest dose modifications being made to the chemotherapy treatment. A total of 37% of patients required a dose modification to the planned chemotherapy schedule. These were usually minor dose modifications or delays, most commonly resulting from 5FU-induced mucosal toxicity. Overall, the patients received 94 and 93% of the planned cisplatin and 5FU, respectively. Four patients were changed to carboplatin as a result of neuro-/nephrotoxicity. Only six (7%) patients received less than the prescribed dose of radiation or a delay of more than 1 week in their RT treatment.

There were no deaths directly related to treatment, though five patients died during or within 30 days of completing treatment due to progressive disease. One patient had a suspected pulmonary embolism but a post mortem was not carried out. Significant toxicities are shown in Table 3

**Table 3:** Significant (WHO grade 3/4) treatment toxicities sustained during chemoradiation

	**Grade 3(%)**	**Grade 4(%)**
*Haematological*		
Neutropaenia	8	2
Anaemia	5	1
		
*Mucositis*		
Oral	12	0
Oesophageal	3	0
		
Palmar–plantar erythema	5	0
		
*Gastrointestinal*		
Nausea	2	0
Diarrhoea	5	0
		
*DVT*		
CVC-related	3	0
Non-CVC-related	2	0
		
Neuropathy	3	0
Lethargy/asthenia	3	1
Other	5	0
		
Total	56	4

CVC=central venous catheter, DVT=deep-vein thrombosis.

. In all, 45 patients (50%) suffered one or more WHO grade 3 or 4 toxicities, being grade 3 in 93% of cases. Haematological toxicity occurred in 16 patients, the majority being neither serious nor associated with admission to hospital. Seven patients required blood transfusion. In 22 cases (37%), patients experienced moderate/severe mucocutaneous toxicity as a consequence of 5FU, which responded to appropriate dose modifications.

In all, 17 patients required nonelective hospitalisation due to treatment complications, most commonly for rehydration as a result of dysphagia, electrolyte disturbance and mucosal toxicity. In addition, 18 patients (20%) required enteral feeding as a result of malignant dysphagia and five patients developed deep venous thrombosis, which required anticoagulation.

During follow-up, nine (10%) patients developed nonmalignant strictures requiring dilatation and four patients developed tracheo-oesophageal fistulae, all of whom had a covered stent successfully placed in the oesophagus. Three patients had a persistent ulcerative oesophagitis and two a temporary radiation pneumonitis, which settled with appropriate treatment.

### Patient survival

As of 31 January 2003, 45 (50%) of patients were alive, 36 with no evidence of disease progression. One patient has been lost to follow-up. The median progression-free survival for the whole group was 18 (9, 38) months.

**Figure 1:**
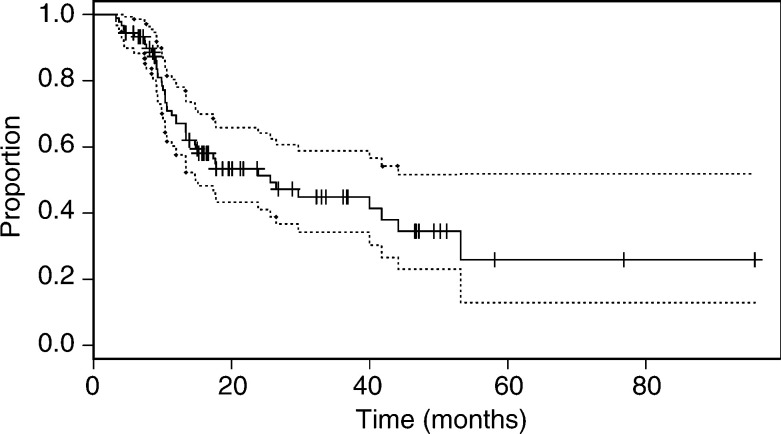
Overall survival of 90 patients receiving definitive chemoradiation for inoperable oesophageal cancer.

**Figure 2:**
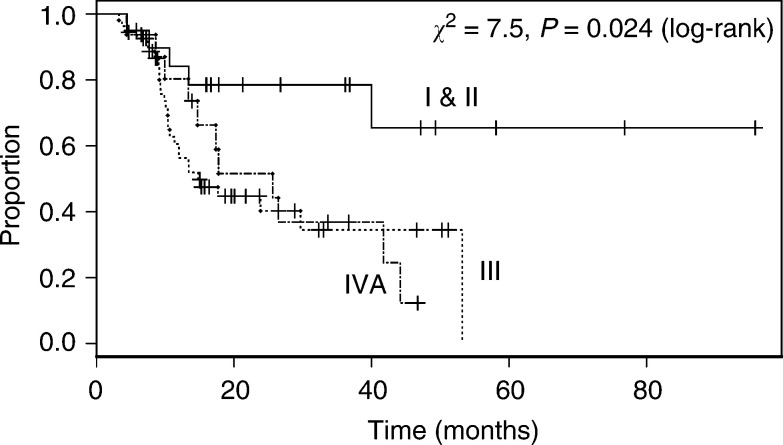
Overall survival of patients according to Group Stage of disease. Stage I and II (T1N0, T2-3N0, T12N1), Stage III (T3N1, T4N0-1), Stage IVa (M1a disease).

**Figure 3:**
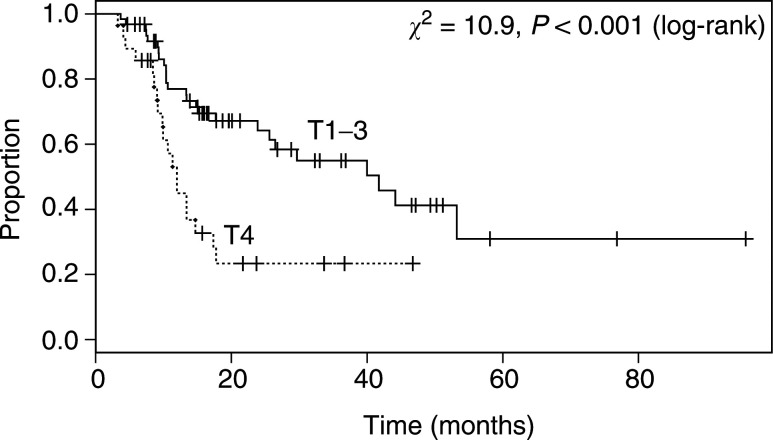
Overall survival of patients according to the stage of primary oesophageal tumour (T stage).

The median overall survival on an intention to treat analysis was 26 (15, >96) months. For the whole series, the 2-, 3- and 5-year survivals were 51% (41, 64), 45% (34, 59) and 26% (13, 52), respectively (Figure 1

). In general, patients fared less well with more advanced disease, and in general the effect of stage was significant (*P*=0.024, Figure 2

). The median survival of 20 patients with stage I or II disease had not been reached (>96 months), 78% (62, 100) being alive after 2 years. However, there was no significant difference in survival between patients with stage III and IVa disease (*P*=0.89; median survival times 15 (11, >53) and 26 (15, >47) months, respectively). In patients with T4 disease, the overall survival was significantly worse than in those with non-T4 (*P*<0.001, Figure 3

).

In 38 patients where the extent of their disease determined that the chance of a complete resection (R0) was too low, the median survival was 11 (10, 42) months, whereas, in the 52 cases where patient-related factors precluded surgery, the survival was significantly better, (*P*<0.001; median survival time 40 (26, >96) months). In the latter group, the 2- and 5-year survivals were 67% (54, 84) and 32% (13, 79), respectively.

In a Cox regression model, none of the covariates age, sex, histological type, the presence of M1a disease (coeliac lymphadenopathy associated with a tumour of the lower third of the oesophagus) and nodal status were significantly associated with outcome (*P*>0.29). The only significant prognostic indicator was the presence of T4 disease, when compared with T1–T3 (hazard ratio=3.21 (1.32, 7.81), *P*=0.01). The reason for not having surgery was excluded from the Cox model because this factor is partly derived from the stage of disease, and, as would be expected, the association between the two was highly significant (*χ*^2^=37, *P*<<0.001). Of those cases deemed to be unsuitable for surgery because of their disease, 66% were T4.

### Patterns of failure

In all, 49 (54%) patients developed progressive disease during the period of study. A total of 17 patients (19%) had radiological or clinical evidence of disease progression at the end of or just after completion of treatment. In a further 21 cases (23%), disease recurrence was confined to locoregional sites, defined as lying within the planned RT target volume. In eight patients, the initial relapse was at a distant site and in three both local and distant disease progression occurred together.

### Second-line treatment

The 49 patients who relapsed were considered for second-line treatment, but in 23 (47%) best supportive care was deemed most appropriate. Of 26 patients who received active second-line palliative therapy, 22 (85%) underwent endoscopic therapy to palliate local disease, this being the placement of an oesophageal stent in 19 (73%) and laser therapy in three. For those patients who had distant metastases as a component of disease relapse, five (42%) underwent second-line chemotherapy. The median survival of patients after disease progression was 5.1 (3.7, 13) months.

## DISCUSSION

In this series, 90 patients with oesophageal cancer who received definitive chemoradiation, having been selected for nonsurgical therapy because of co-morbidity or locally advanced disease, had a median survival of 26 months, 51 and 26% being alive 2 and 5 years, respectively, after diagnosis.

These results compare favourably with other published series evaluating the use of chemoradiation for oesophageal cancer (Coia *et al*, 1991; Cooper *et al*, 1999; Kaneko *et al*, 2003). The most important of these, RTOG 85-01, was a US Intergroup randomised controlled trial comparing chemoradiation with RT alone (Cooper *et al*, 1999). In the final report, the 5-year survival of patients who underwent a similar schedule of chemoradiation was 27%. The majority of patients in this study had squamous carcinoma, and it was not very clear how patients were selected for nonsurgical treatment. In addition, staging investigations would be considered suboptimal by today's standards.

In the US Intergroup Study, the two cycles of nonconcurrent chemotherapy were given after chemoradiation, and only about half the patients were able to complete this phase of therapy. By giving these cycles in the neoadjuvant setting, treatment can start straight away and patient's dysphagia is often improved prior to the RT. It was hoped in our current protocol that this would be more a more tolerable strategy for delivering additional systemic therapy. In addition, protracted venous infusional (PVI) 5FU was used. This has been shown to be superior to bolus treatments in other cancers (Meta-analysis Group, 1998) and optimises the radio-sensitising properties of this drug, which has a short half-life. Although, in our study, chemotherapy modifications were required in 37% of patients, the great majority of these were due to troublesome rather than life-threatening toxicities, and indeed 93% of the overall planned chemotherapy dose was delivered.

In a similar number of patients, Sykes *et al* (1998) demonstrated that, in carefully selected patients, long-term survival can be achieved using the radiation-based therapy. As in our series, patients were selected because they were not suitable for surgery. However, although EUS was not available, the majority of patients had tumours less than 5 cm in length, which is associated with a significantly reduced risk of mediastinal lymphadenopathy, and over 90% of cases had squamous carcinomas. There were significantly more adenocarcinomas in our series and the mean length of tumour was 6.9 cm (mean RT field length 14.1 cm). This makes direct comparisons difficult, but the better outcome in median, 2-, 3- and 5-year survival may be due to the superiority of chemoradiation over RT alone.

How do these results compare to surgical series? There have recently been two large multicentred randomised controlled trials evaluating the role of chemotherapy prior to surgery. The UK MRC OE02 trial demonstrated a clear benefit for neoadjuvant treatment (Medical Research Council Oesophageal Cancer Working Group, 2002). The overall survival at 2 years was significantly increased from 35 to 44%, and median survival from 13.4 to 17.6 months in favour of preoperative chemotherapy. In the US Intergroup 0116 trial, there was no statistical difference in overall survival, 2-year and median survivals being approximately 35% and 15.5 months, respectively (Kelsen *et al*, 1998). These are probably the most reliable results to benchmark outcome from modern surgical intervention. We report a 2-year survival of 51% and a median survival of 26 months. While it is necessary to be cautious when comparing retrospective series with randomised trial data, it is worth noting that, in our series, all patients were considered not suitable candidates for surgery because of adverse factors relating to the patients or their disease. This group of patients therefore fall into a poor prognostic group; indeed, previously many would have been considered only for palliative therapy.

**Figure 4:**
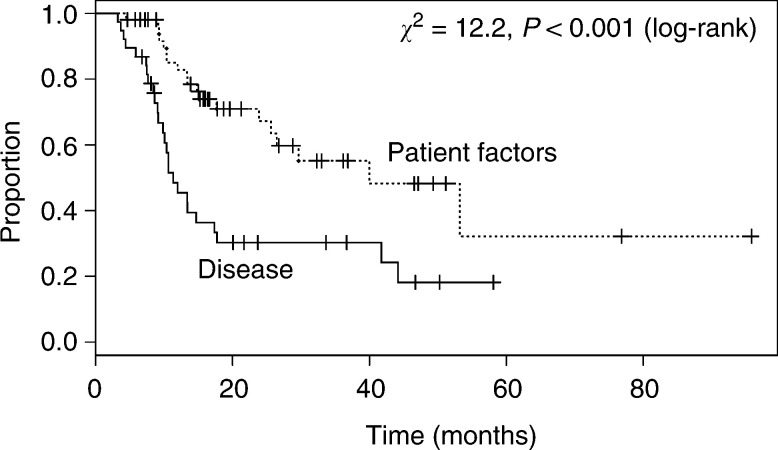
Overall survival of patients according to the predominant reason given for not undergoing surgery. Patient factors=Co morbidity, performance status and patient choice.

There was a highly significant difference in outcome between those who did not receive surgery due to characteristics relating to the patients, for example, co-morbidity, performance status, patient choice, etc, compared with factors relating to their disease, for example, invasion of mediastinal organs, extensive lymphadenopathy, etc., (*P*<0.001, Figure 4

). Many patients with oesophageal cancer have chronic co-morbidity because of shared aetiological factors, namely chronic obstructive pulmonary disease, ischaemic heart disease and age. While these may increase the risks of surgery prohibitively, they need not be immediately life threatening. Contrasting with this, those patients considered unresectable usually had disease overtly infiltrating other mediastinal organs, namely the aorta, trachea or pleura, or extensive/bulky lymphadenopathy. Although these patients may have some initial palliative benefit from definitive CRT, it will rarely achieve long-term disease control. Indeed, Cox regression analysis indicated nonindependence between tumour stage and the reason for not having surgery, in terms of prognosis.

As expected, advancing stage of disease was generally associated with a poorer outcome and patients with stage I or II disease had a significantly better prognosis than patients with stage III and IV. The reason why stage IVa (any T, any N, M1a) patients did not show a significantly reduced survival rate than patients with stage III (T3 N1 or T4 any N, M0) disease may be that the latter patients had on average more bulky disease than the former. In this cohort, all those with Stage IVa disease had nonbulky coeliac nodal involvement with cancer in the lower on-third oesophagus (M1a disease). This extent of disease may have a more favourable outcome with or without therapeutic intervention than patients with stage III disease not receiving surgery. Cox regression showed that T4 disease had a greater impact on outcome than the presence of M1a, and in stage III cases the proportion of T4 tumours was higher (68%) than in stage IVa cases (32%).

Although chemoradiation is considered to be superior in terms of local control and survival over RT alone, it is associated with increased toxicity (Cooper *et al*, 1999; Wong *et al*, 2003). In the current series, apart from admission to hospital for ‘hotel’ needs and enteral feeding for malignant dysphagia, 19% of patients required admission to hospital for treatment-related toxicity. The majority of these admissions were for intravenous rehydration as a result of oral mucositis, oesophagitis and diarrhoea. As well as this, some patients felt extremely weak and tired towards the end of treatment. This complex treatment should only be undertaken where there is an experienced team. In our centre, patients benefit from an upper GI specialist nurse, a dedicated dietician, and a radiographer familiar with combined chemoradiation regimens.

As well as the encouraging results with respect to overall survival, it is also particularly encouraging that, several weeks after completing this treatment, patients frequently feel very well and most notably their dysphagia has usually resolved. This, and other aspects of quality of life, is the subject of ongoing research by the South East Wales Oesophago-Gastric Unit.

What proportion of patients may be suitable for this regimen? In 1995, an audit of all cases diagnosed with oesophagogastric cancer in Wales found the incidence of oesophageal cancer to be 11/100 000 (Pye *et al*, 2001). Based upon this data, Velindre Cancer Centre serving South East Wales with a population of 1.48 million people would expect to see 165 patients per year with this disease. In 2001, the most recent complete annual data available, 152 patients diagnosed with oesophageal cancer were referred for consideration of treatment. Of these, 86 patients were treated with palliative intent. Of the 66 cases that were thought suitable for potentially curative therapy, the distribution of treatment modalities used is shown in Table 4

**Table 4:** Distribution of treatment modalities used for 66 cases of oesophageal cancer treated with curative intent in 2001

**Treatment modality**	**Number (%)**
*Surgical-based therapy*	
Surgery alone	5 (7.5%)
Surgery+preoperative chemotherapy	10 (15%)
Surgery+preoperative chemoradiation	12 (18%)
	
*Radiotherapy based therapy*	
Radiotherapy alone	6 (9%)
Chemoradiation	33 (50%)
	
Total	66 #

. On the basis of the most recent data available, this therapy was given to 21% of all patients with oesophageal cancer and 50% of those being treated with curative intent. It is possible that a greater proportion of patients would benefit from this treatment, particularly those patients currently being treated with palliative intent or some currently receiving surgical based treatment.

In the United States, results from the ‘Patterns of Care’ report reflect that chemoradiation is already a standard treatment option for patients with nonmetastatic disease (Coia *et al*, 2000). In all, 45% of the patients received CRT as their sole therapy, and in 32.8% this was with a RT dose of ⩾50 Gy. With increasing use of this treatment, it is essential to optimise the delivery of this therapeutic approach. Despite the encouraging survival rate in this report, disease progression at the site of original disease is the predominant pattern of treatment failure. Of 49 patients who developed progressive disease, there was a failure to achieve local control at the end of treatment in 17 cases and a further 21 developed evidence of local disease progression during follow-up. Therefore, locoregional disease was a component of treatment failure in 84% of cases, whereas metastatic disease was a component in 22%. The overall local failure rate of 42% is similar to other chemoradiation studies (Minsky *et al*, 2002), but possibly higher than that seen after surgery (Kelsen *et al*, 1998; Dresner *et al*, 2000).

Undoubtedly, the way to improve outcomes is through well-conducted research. This disease site has been generally been poor in recruitment of patients into clinical studies, whereas chemoradiation trials in other cancer sites such as anal cancer demonstrate that high rates of accrual can be obtained (Anal Cancer Trial Working Party, 1996). Evidence-based ‘best practice’ protocols need to evolve through collaboration between specialist centres in the UK, where it can be shown that this complex therapy can be delivered safely. Changing body contours and tissue densities together with the close proximity of important normal tissue structures make tumours of the oesophagus a challenging disease in which to deliver a homogenous dose of radiation, and therefore a prime tumour site in which to test innovative technologies such as intensity-modulated radiotherapy (IMRT). There is evidence for a radiation dose effect in this disease (Geh *et al*, 2000), though attempts to increase the dose have not yet led to an improved outcome (Minsky *et al*, 2002).

Capecitabine and oxaliplatin are being tested in advanced oesophagogastric cancer and would certainly provide a more convenient and hopefully more active systemic regimen. Irinotecan and taxanes have also shown high activity in Upper GI malignant disease, together with promising radiosensitising properties.

These data show that definitive chemoradiation is feasible and tolerable for 50% of patients with oesophageal cancer, treated with potentially curative therapy. It supports other evidence that this modality is the treatment of choice in patients unsuitable for surgery on the basis of co-morbidity or locally advanced disease. It also suggests that it should be compared in a prospective study to a surgically based treatment strategy.

## References

[bib1] Anal Cancer Trial Working Party (1996) Epidermoid anal cancer: results from the UKCCCR randomised trial of radiotherapy alone versus radiotherapy, 5-fluorouracil, and mitomycin. UK Co-ordinating Committee on Cancer Research. Lancet 348: 1049–1054 8874455

[bib2] Blazeby JM, Farndon JR, Donovan J, Alderson D (2000) A prospective longitudinal study examining the quality of life of patients with esophageal carcinoma. Cancer 88: 1781–1787 10760752

[bib3] Bowrey D, Clark G, Roberts SA, Hawthorne AB, Maughan TS, Williams GT, Carey PD (1999) Serial endoscopic ultrasound assessment of response to chemoradiotherapy for carcinoma of the esophagus. J Gastrointest Surg 3: 462–467 10482701 10.1016/s1091-255x(99)80098-5

[bib4] Coia LR, Engstrom PE, Paul AR, Stafford PM, Hanks GE (1991) Longterm results of infusional 5-FU, mitomycin-C, and radiation as primary management of esophageal carcinoma. Int J Radiat Oncol Biol Phys 20: 29–36 1704362 10.1016/0360-3016(91)90134-p

[bib5] Coia LR, Minsky BD, Berkey BA, John MJ, Haller D, Landry J, Pisansky TM, Willett CG, Hoffman JP, Owen JB, Hanks GE (2000) Outcome of patients receiving radiation for cancer of the esophagus: results of the 1992–1994 patterns of care study. J Clin Oncol 18: 455–462 10653860 10.1200/JCO.2000.18.3.455

[bib6] Cooper JS, Guo MD, Herskovic A, Macdonald JS, Martenson Jr JA, Al-Sarraf M, Byhardt R, Russell AH, Beitler JJ, Spencer S, Asbell SO, Graham MV, Leichman LL (1999) Chemoradiotherapy of locally advanced esophageal cancer: long-term follow-up of a prospective randomized trial (RTOG 85-01). Radiation Therapy Oncology Group. JAMA 281: 1623–1627 10235156 10.1001/jama.281.17.1623

[bib7] Dresner SM, Griffin SM (2000) Pattern of recurrence following radical oesophagectomy with two-field lymphadenectomy. Br J Surg 87: 1426–1433 11044172 10.1046/j.1365-2168.2000.01541.x

[bib8] Fok M, Law SY, Wong J (1994) Operable esophageal carcinoma: current results from Hong Kong. World J Surg 18: 355–360 8091775 10.1007/BF00316814

[bib9] Geh JI, Bond SJ, Bentzen SM, Glynne-Jones R (2000) Preoperative chemoradiotherapy in esophageal cancer: evidence of dose response. Proc Annu Meet Am Soc Clin Oncol 19: A958

[bib10] Kaneko K, Ito H, Konishi K, Kurahashi T, Ito T, Katagiri A, Yamanoto T, Kitahara T, Mizutani Y, Ohtsu A, Mitamura K (2003) Definitive chemoradiotherapy for patients with malignant stricture due to T3 or T4 squamous carcinoma of the oesophagus. Br J Cancer 88: 18–24 12556953 10.1038/sj.bjc.6600684 PMC2376792

[bib11] Kelsen DP, Ginsberg R, Pajak TF, Sheahan DG, Gunderson L, Mortimer J, Estes N, Haller DG, Ajani J, Kocha W, Minsky BD, Roth JA (1998) Chemotherapy followed by surgery compared with surgery alone for localized esophageal cancer. N Engl J Med 339: 1979–1984 9869669 10.1056/NEJM199812313392704

[bib12] Medical Research Council Oesophageal Cancer Working Group (2002) Surgical resection with or without preoperative chemotherapy in oesophageal cancer: a randomised controlled trial. Lancet 359: 1727–1733 12049861 10.1016/S0140-6736(02)08651-8

[bib13] Meta-analysis Group In Cancer (1998) Efficacy of intravenous continuous infusion of fluorouracil compared with bolus administration in advanced colorectal cancer. J Clin Oncol 16: 301–308 9440757 10.1200/JCO.1998.16.1.301

[bib14] Minsky BD, Pajak TF, Ginsberg RJ, Pisansky TM, Martenson J, Komaki R, Okawara G, Rosenthal SA, Kelsen DP (2002) INT 0123 (Radiation Therapy Oncology Group 94-05) phase III trial of combined-modality therapy for esophageal cancer: high-dose versus standard-dose radiation therapy. J Clin Oncol 20: 1167–1174 11870157 10.1200/JCO.2002.20.5.1167

[bib15] NHS Executive (2001) Guidance on Commissioning Cancer Services – improving outcomes in upper gastro-intestinal cancers. (NHS Executive Catalogue Number 23943)

[bib16] Pye JK, Crumplin MK, Charles J, Kerwat R, Foster ME, Biffin A (2001) Hospital clinicians in Wales. One-year survey of carcinoma of the oesophagus and stomach in Wales. Br J Surg 88: 278–285 11167881 10.1046/j.1365-2168.2001.01655.x

[bib17] Scottish Audit of Gastric and Oesophageal Cancer Steering Group (2002) Scottish Audit of Gastric and Oesophageal Cancer Report 1997–2000: A Prospective Audit. (http://www.show.scot.nhs.uk/crag/)

[bib18] Siewert JR, Stein HJ (1998) Classification of adenocarcinoma of the oesophagogastric junction. Br J Surg 85: 1457–1459 9823902 10.1046/j.1365-2168.1998.00940.x

[bib19] Sykes AJ, Burt PA, Slevin NJ (1998) Radical radiotherapy for carcinoma of the oesophagus: an effective alternative to surgery. Radiother Oncol 48: 15–21 9756167 10.1016/s0167-8140(98)00037-1

[bib20] Walsh T, Noonan N, Hollywood D (1996) A comparison of multimodal therapy and surgery for oesophageal adenocarcinoma. N Engl J Med 335: 462–467 8672151 10.1056/NEJM199608153350702

[bib21] Wong RKS, Malthaner RA, Zuraw L, Bryan Rumble R, The Cancer Care Ontario Practice Guidelines Initiative Gastrointestinal Cancer Disease Site Group (2003) Combined modality radiotherapy and chemotherapy in nonsurgical management of localized carcinoma of the esophagus: a practice guideline. Int Radiat Oncol Biol Phys 55: 930–942 10.1016/s0360-3016(02)04278-5 12605971

